# Supplementary material for: A mitochondrial genome phylogeny of voles and lemmings (Rodentia: Arvicolinae): Evolutionary and taxonomic implications
Source: PLoS One. 2021 Nov 19;16(11):e0248198. doi: 10.1371/journal.pone.0248198 (PMC8604340; doi:10.1371/journal.pone.0248198)

S1 Fig. Mapping of raw Illumina reads of *Lemmiscus curtatus* against the reference mitochondrial genome of *Mynomes ochrogaster* using Geneious Prime.

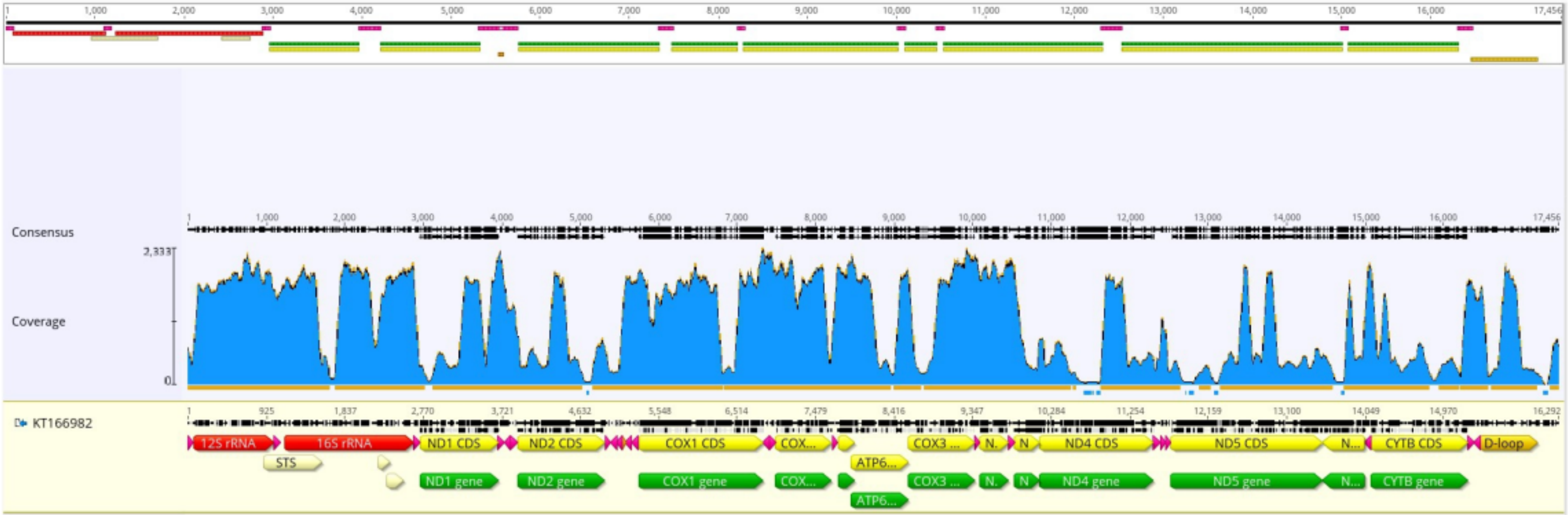

Supplement: S1 Fig — (PDF) [file pone.0248198.s001.pdf]
