# Supplementary material for: A mitochondrial genome phylogeny of voles and lemmings (Rodentia: Arvicolinae): Evolutionary and taxonomic implications
Source: PLoS One. 2021 Nov 19;16(11):e0248198. doi: 10.1371/journal.pone.0248198 (PMC8604340; doi:10.1371/journal.pone.0248198)

S2 Fig. Nucleotide misincorporations at 5'-termini (A) and 3'-termini (B) of the *Lemmiscus curtatus* calculated using mapDamage

All possible misincorporations are plotted in gray, except for guanine to adenine (G>A, blue lines) and cytosine to thymine (C>T, red lines).

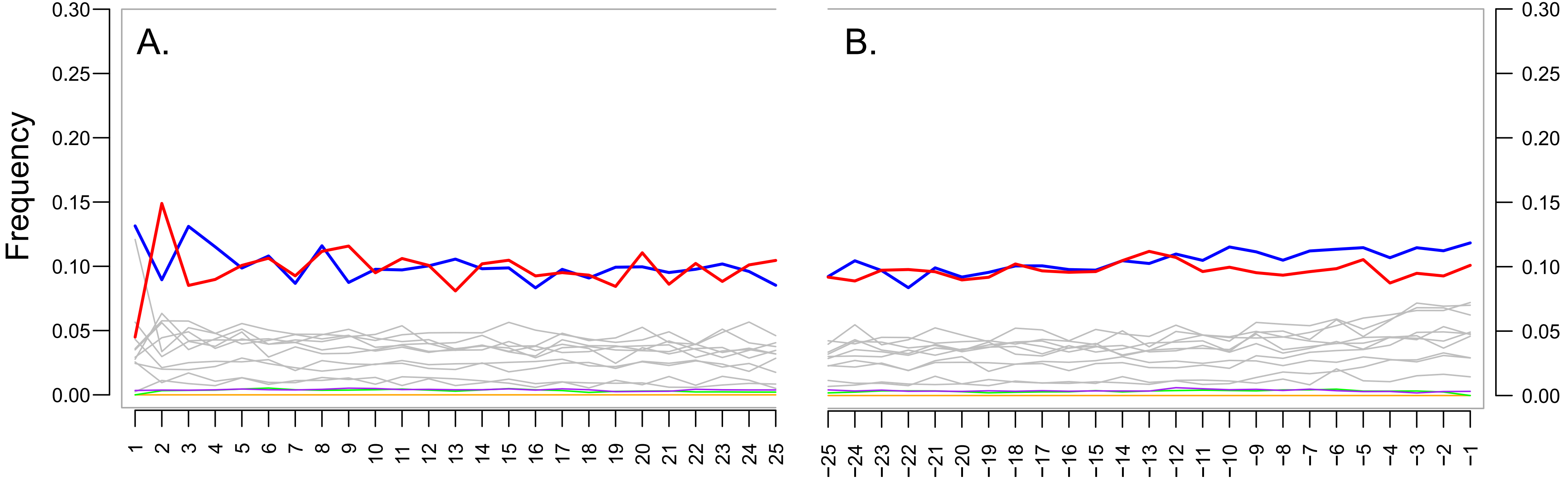

Supplement: S2 Fig — Nucleotide misincorporations at 5’-termini (A) and 3’-termini (B) of the Lemmiscus curtatus calculated using mapDamage. All possible misincorporations are plotted in gray, except for guanine to adenine (G>A, blue lines) and cytosine to thymine (C>T, red lines). (PDF) [file pone.0248198.s002.pdf]
