# Supplementary figures and images for: A mitochondrial genome phylogeny of voles and lemmings (Rodentia: Arvicolinae): Evolutionary and taxonomic implications
Source: PLoS One. 2021 Nov 19;16(11):e0248198. doi: 10.1371/journal.pone.0248198 (PMC8604340; doi:10.1371/journal.pone.0248198)

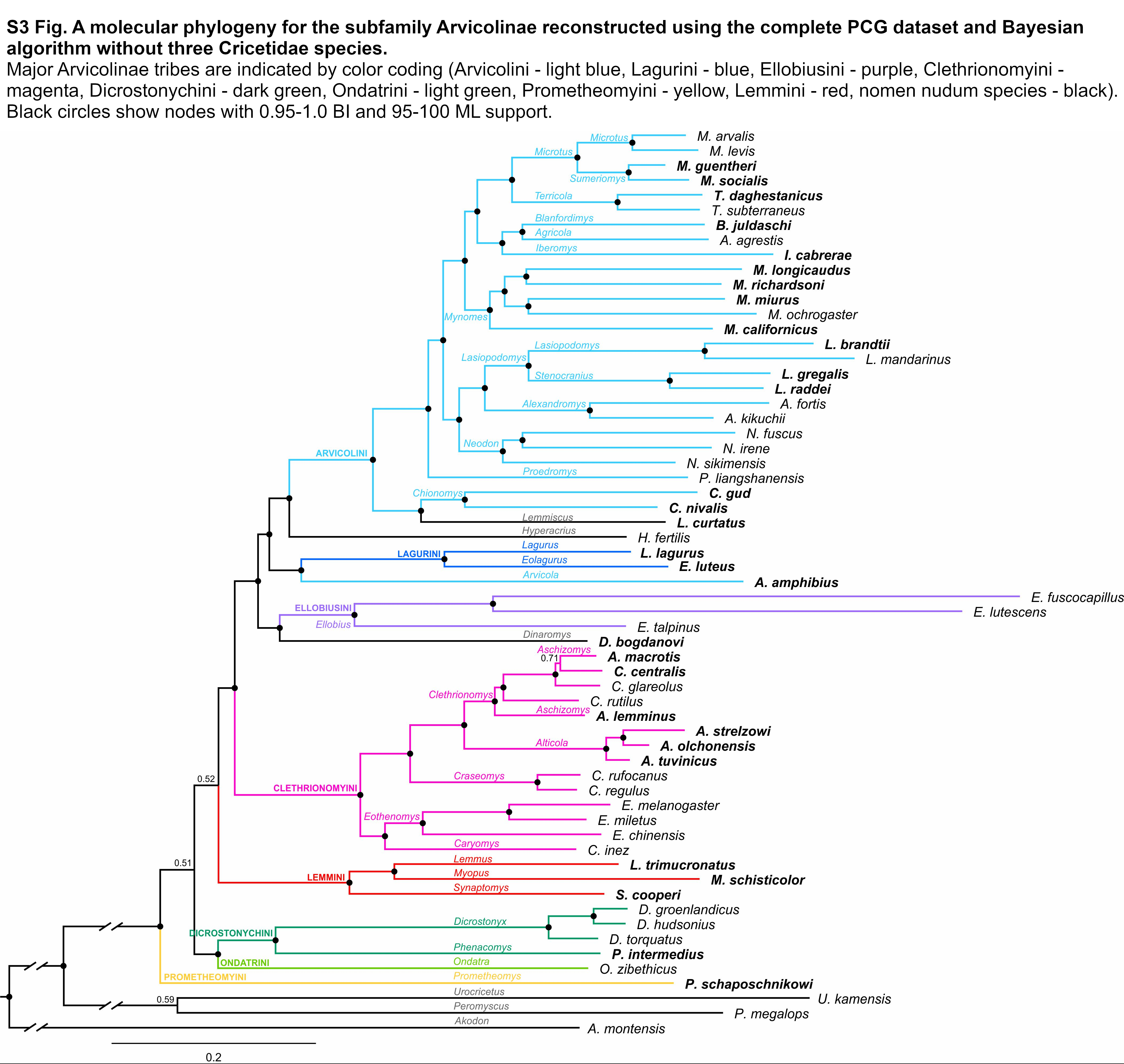

Supplement: S3 Fig — Major Arvicolinae tribes are indicated by color coding (Arvicolini—light blue, Lagurini—blue, Ellobiusini—purple, Clethrionomyini—magenta, Dicrostonychini—dark green, Ondatrini—light green, Prometheomyini—yellow, Lemmini—red, nomen nudum species—black). Black circles show nodes with 0.95–1.0 BI and 95–100 ML support. (JPG) [file pone.0248198.s003.jpg]
