# Supplementary material for: A mitochondrial genome phylogeny of voles and lemmings (Rodentia: Arvicolinae): Evolutionary and taxonomic implications
Source: PLoS One. 2021 Nov 19;16(11):e0248198. doi: 10.1371/journal.pone.0248198 (PMC8604340; doi:10.1371/journal.pone.0248198)
Supplement: S1 File — Major Arvicolinae tribes are indicated by color coding (Arvicolini—light blue, Lagurini—blue, Ellobiusini—purple, Clethrionomyini—magenta, Dicrostonychini—dark green, Ondatrini—light green, Prometheomyini—yellow, Lemmini—red, nomen nudum species—black). Bayesian topology was used to plot the tree for the complete 13 PCGs dataset. Node labels display the following supports: BI complete / BI RY-coded 3rd codon position / ML complete / ML RY-coded 3rd codon position. Black circles show nodes with 0.95–1.0 BI and 95–100 ML support. For each of the PCGs, maximum likelihood topology is given, node labels display ultrafast ML bootstrap above 50%. Saturation plots are indicated on the side insets, where colors mark the following partitions: 1st codon position transitions (ts)—brown, 1st transversions (tv)—red, 2nd ts—blue, 2nd tv—green, 3rd ts—pink, 3rd tv—black. (PDF) [file pone.0248198.s011.pdf]

S1 File. A molecular phylogeny for the subfamily Arvicolinae reconstructed using the complete PCG dataset and each of the 13 individual genes with the corresponding saturation plots indicated.
Major Arvicolinae tribes are indicated by color coding (Arvicolini - light blue, Lagurini - blue, Ellobiusini - purple, Clethrionomyini - magenta, Dicrostonychini - dark green, Ondatrini - light green, Prometheomyini - yellow, Lemmini - red, nomen nudum species - black).
Bayesian topology was used to plot the tree for the complete 13 PCGs dataset. Node labels display the following supports: BI complete / BI RY-coded 3rd codon position / ML complete / ML RY-coded 3rd codon position. Black circles show nodes with 0.95-1.0 BI and 95-100 ML support.
For each of the PCGs, maximum likelihood topology is given, node labels display ultrafast ML bootstrap above 50%. Saturation plots are indicated on the side insets, where colors mark the following partitions: 1st codon position transitions (ts) - brown, 1st transversions (tv) - red, 2nd ts - blue, 2nd tv - green, 3rd ts - pink, 3rd tv - black.

13 PCG dataset

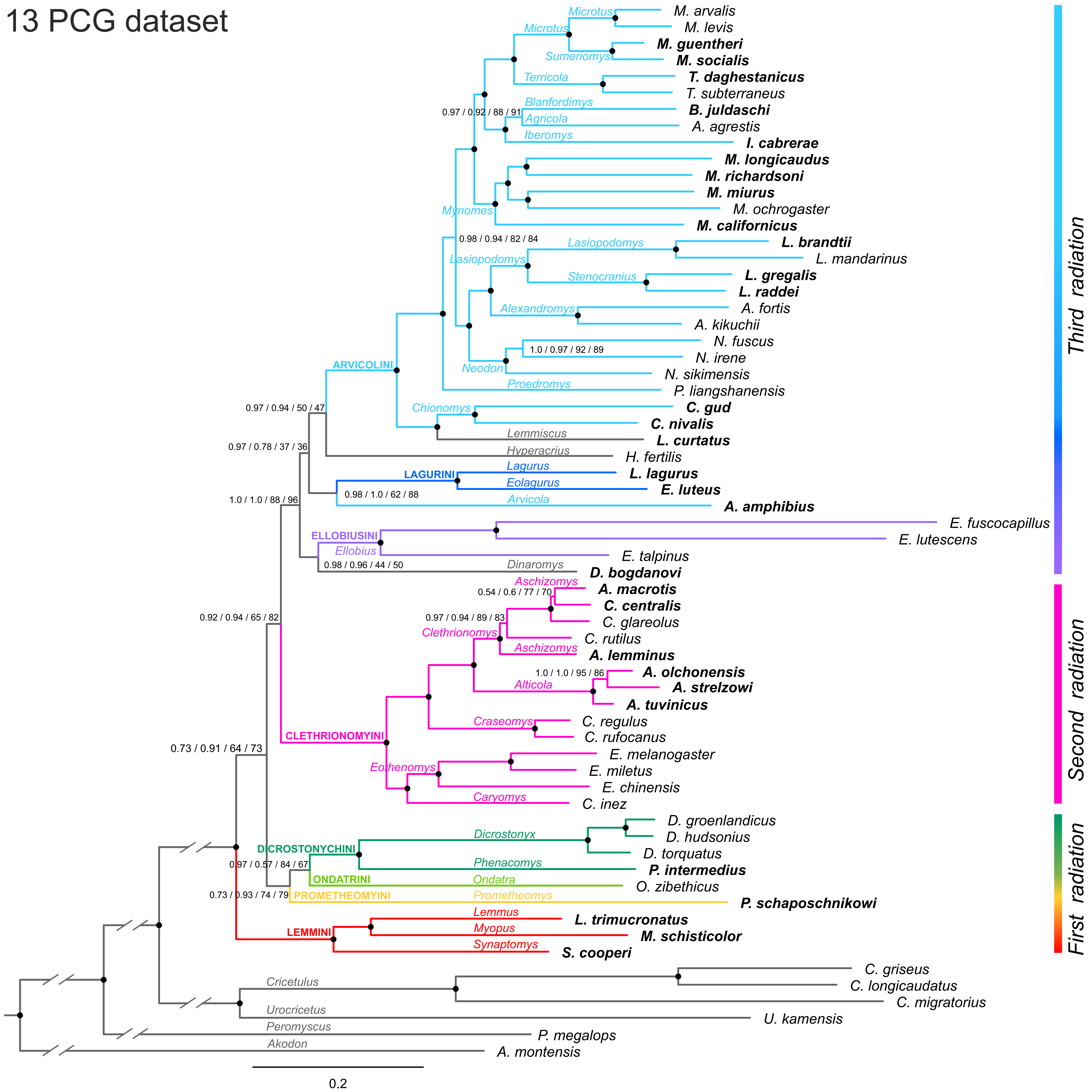

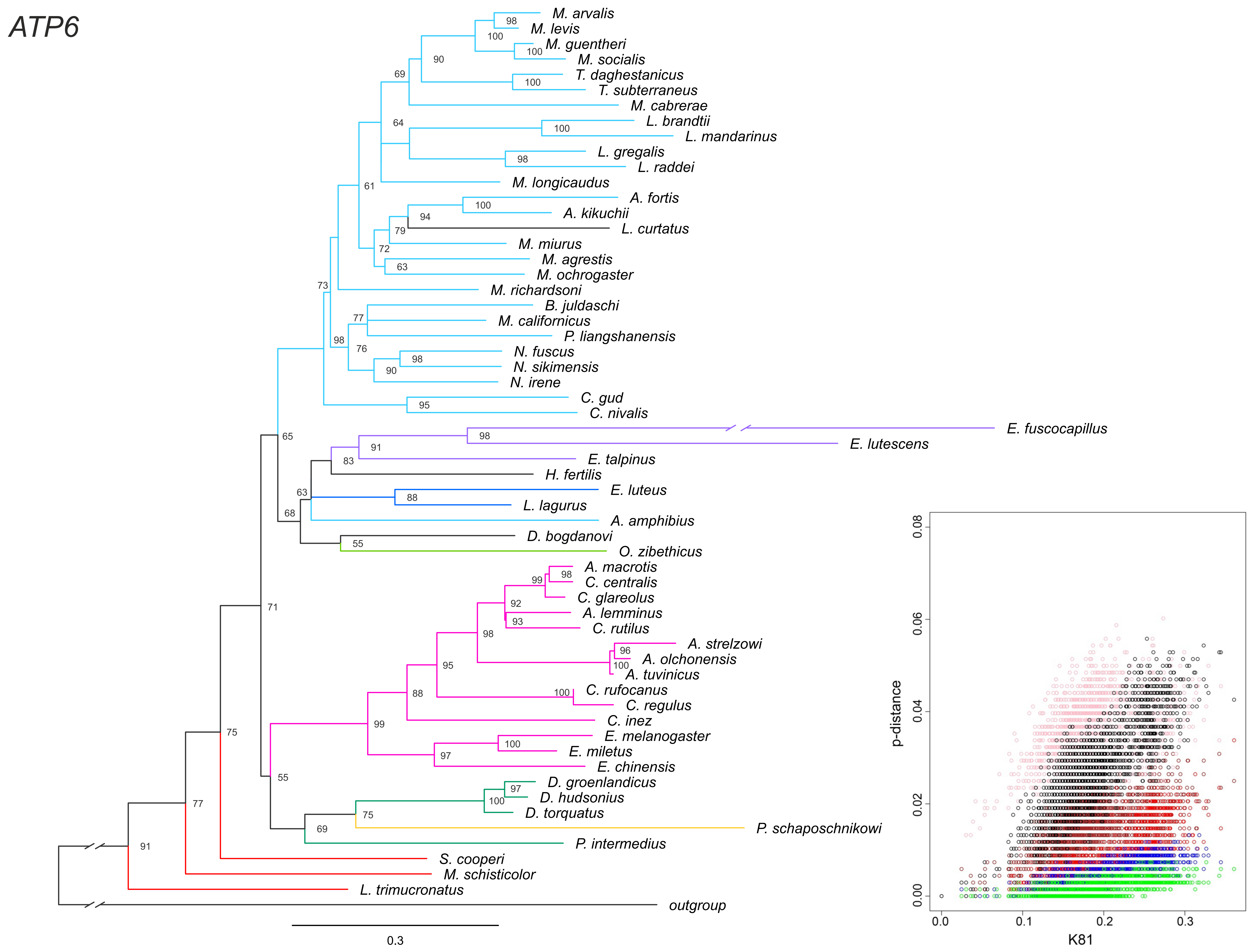

ATP6
M. arvalis
M. levis
M. guentheri
M. socialis
T. daghestanicus
T. subterraneus
M. cabrerae
L. brandtii
L. mandarinus
L. gregalis
L. raddei
M. longicaudus
A. fortis
A. kikuchii
L. curtatus
M. miurus
M. agrestis
M. ochrogaster
M. richardsoni
B. juldaschi
M. californicus
P. liangshanensis
N. fuscus
N. sikimensis
N. irene
C. gud
C. nivalis
E. fuscocapillus
E. lutescens
E. talpinus
H. fertilis
E. luteus
L. lagurus
A. amphibius
D. bogdanovi
O. zibethicus
A. macrotis
C. centralis
C. glareolus
A. lemminus
C. rutilus
A. strelzowi
A. olchonensis
A. tuvinicus
C. rufocanus
C. regulus
C. inez
E. melanogaster
E. miletus
E. chinensis
D. groenlandicus
D. hudsonius
D. torquatus
P. schaposchnikowi
P. intermedius
S. cooperi
M. schisticolor
L. trimucronatus
outgroup
0.3
p-distance
K81
0.00
0.02
0.04
0.06
0.08
0.0
0.1
0.2
0.3

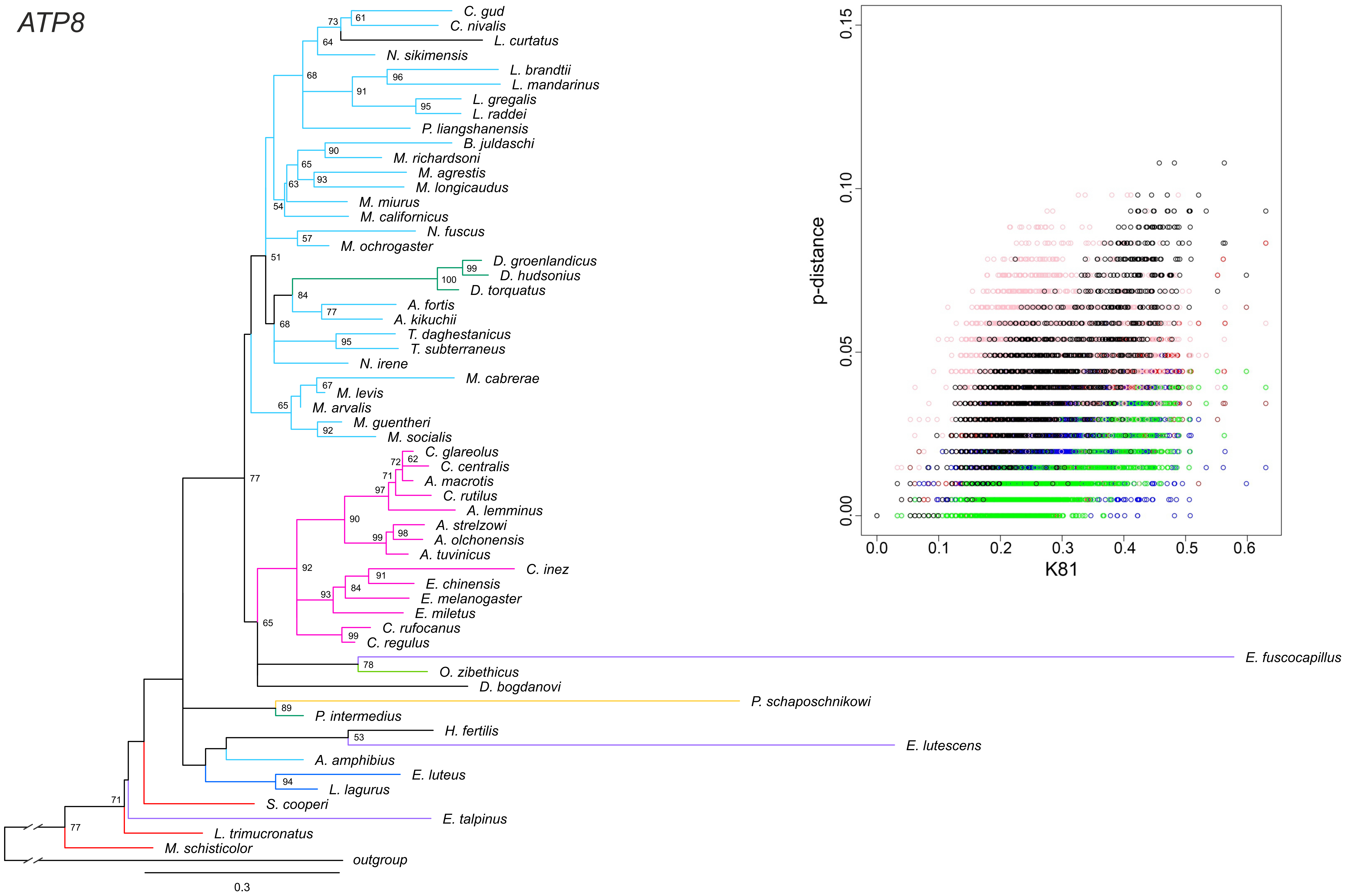

ATP8
C. gud
C. nivalis
L. curtatus
N. sikimensis
L. brandtii
L. mandarinus
L. gregalis
L. raddei
P. liangshanensis
B. juldaschi
M. richardsoni
M. agrestis
M. longicaudus
M. miurus
M. californicus
N. fuscus
M. ochrogaster
D. groenlandicus
D. hudsonius
D. torquatus
A. fortis
A. kikuchii
T. daghestanicus
T. subterraneus
N. irene
M. cabrerae
M. levis
M. arvalis
M. guentheri
M. socialis
C. glareolus
C. centralis
A. macrotis
C. rutilus
A. lemminus
A. strelzowi
A. olchonensis
A. tuvinicus
C. inez
E. chinensis
E. melanogaster
E. miletus
C. rufocanus
C. regulus
E. fuscocapillus
O. zibethicus
D. bogdanovi
P. schaposchnikowi
P. intermedius
H. fertilis
E. lutescens
A. amphibius
E. luteus
L. lagurus
S. cooperi
E. talpinus
L. trimucronatus
M. schisticolor
outgroup
0.3
p-distance
K81
0.00
0.05
0.10
0.15
0.0
0.1
0.2
0.3
0.4
0.5
0.6

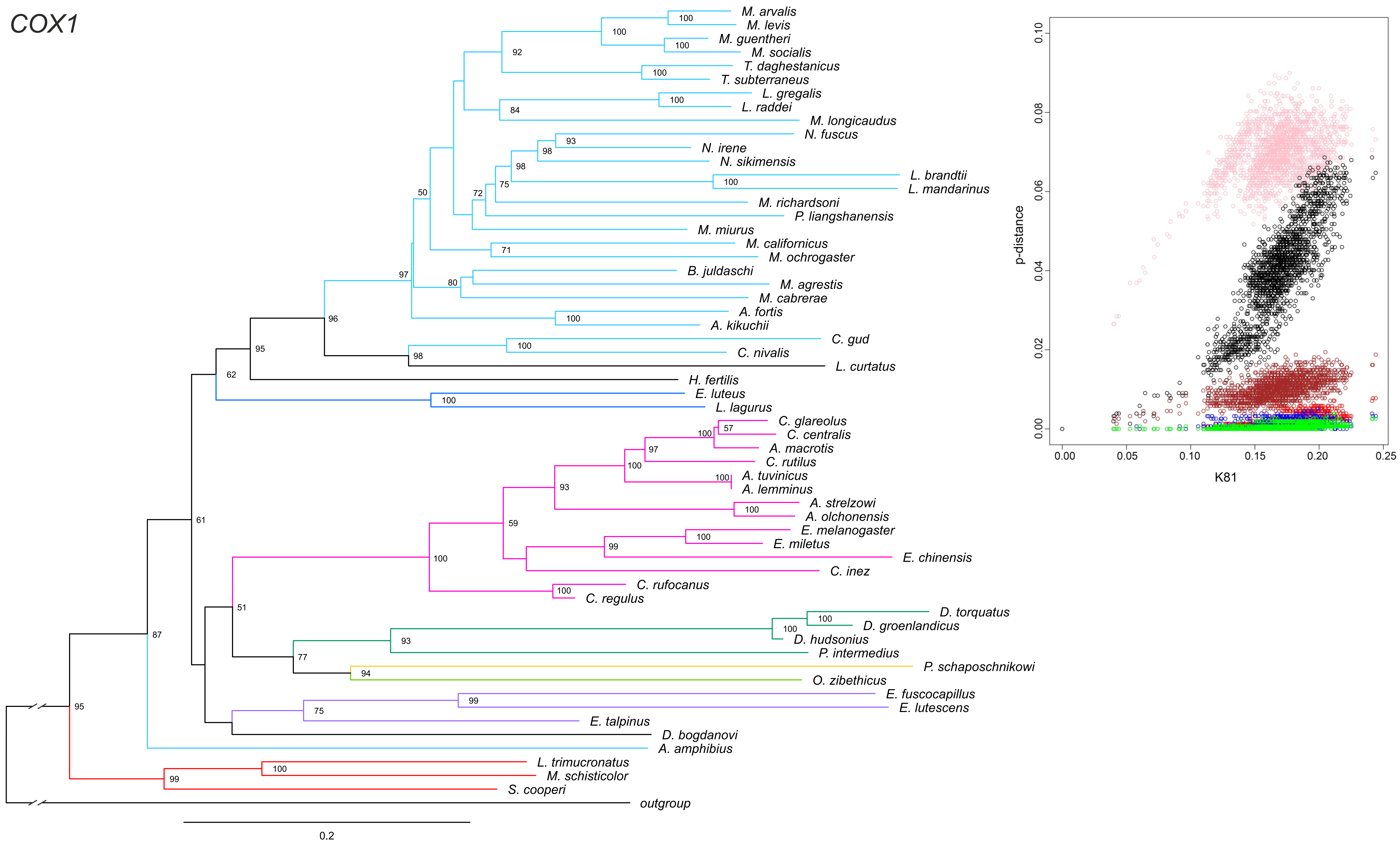
COX1
M. arvalis
M. levis
M. guentheri
M. socialis
T. daghestanicus
T. subterraneus
L. gregalis
L. raddei
M. longicaudus
N. fuscus
N. irene
N. sikimensis
L. brandtii
L. mandarinus
M. richardsoni
P. liangshanensis
M. miurus
M. californicus
M. ochrogaster
B. juldaschi
M. agrestis
M. cabrerae
A. fortis
A. kikuchii
C. gud
C. nivalis
L. curtatus
H. fertilis
E. luteus
L. lagurus
C. glareolus
C. centralis
A. macrotis
C. rutilus
A. tuvinicus
A. lemminus
A. strelzowi
A. olchonensis
E. melanogaster
E. miletus
E. chinensis
C. inez
C. rufocanus
C. regulus
D. torquatus
D. groenlandicus
D. hudsonius
P. intermedius
P. schaposchnikowi
O. zibethicus
E. fuscocapillus
E. lutescens
E. talpinus
D. bogdanovi
A. amphibius
L. trimucronatus
M. schisticolor
S. cooperi
outgroup
0.2
p-distance
K81
0.00
0.02
0.04
0.06
0.08
0.10
0.05
0.15
0.20
0.25

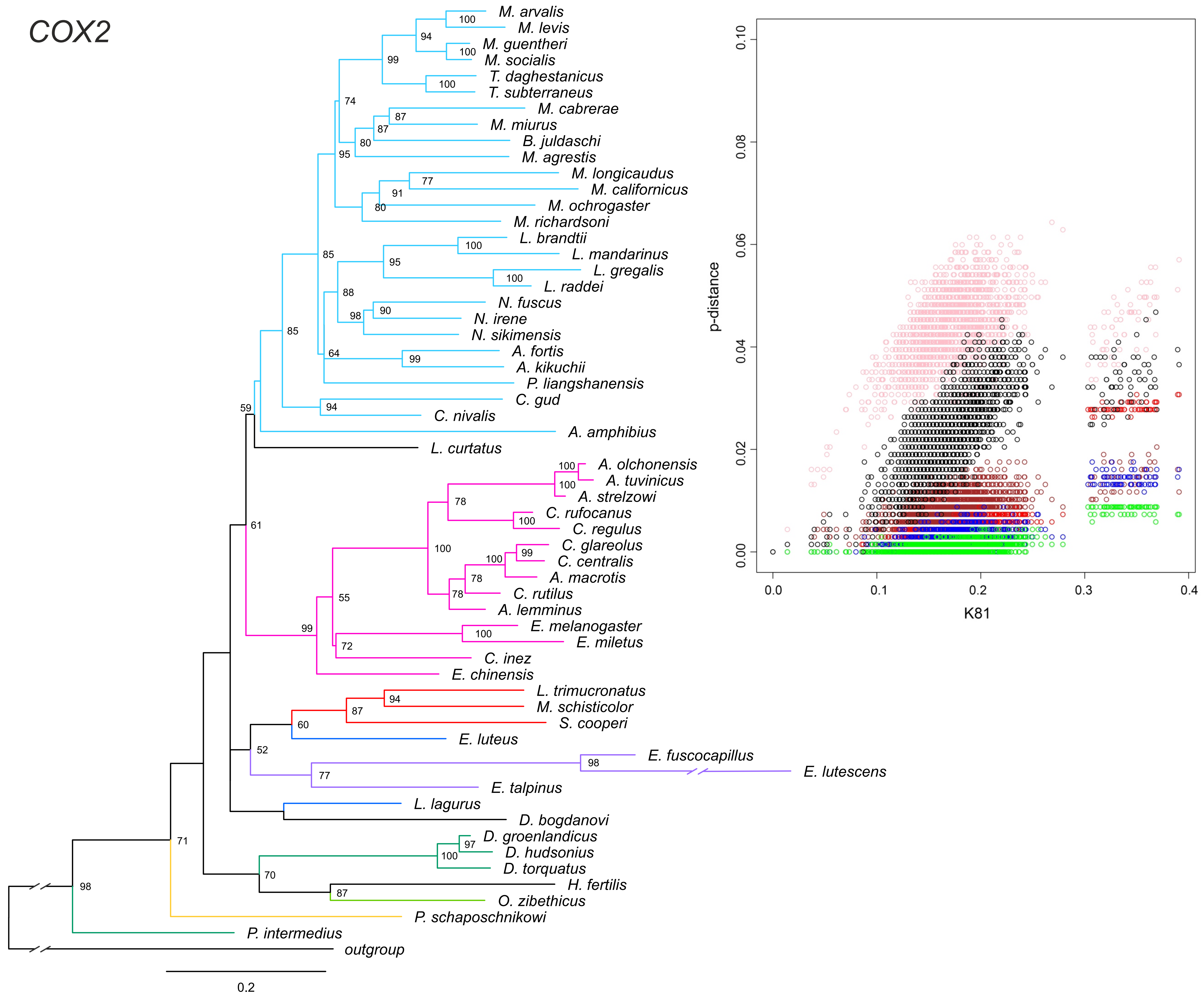

COX2
M. arvalis
M. levis
M. guentheri
M. socialis
T. daghestanicus
T. subterraneus
M. cabrerae
M. miurus
B. juldaschi
M. agrestis
M. longicaudus
M. californicus
M. ochrogaster
M. richardsoni
L. brandtii
L. mandarinus
L. gregalis
L. raddei
N. fuscus
N. irene
N. sikimensis
A. fortis
A. kikuchii
P. liangshanensis
C. gud
C. nivalis
A. amphibius
L. curtatus
A. olchonensis
A. tuvinicus
A. strelzowi
C. rufocanus
C. regulus
C. glareolus
C. centralis
A. macrotis
C. rutilus
A. lemminus
E. melanogaster
E. miletus
C. inez
E. chinensis
L. trimucronatus
M. schisticolor
S. cooperi
E. luteus
E. fuscocapillus
E. lutescens
E. talpinus
L. lagurus
D. bogdanovi
D. groenlandicus
D. hudsonius
D. torquatus
H. fertilis
O. zibethicus
P. schaposchnikowi
P. intermedius
outgroup
0.2
p-distance
0.00
0.02
0.04
0.06
0.08
0.10
K81
0.0
0.1
0.2
0.3
0.4

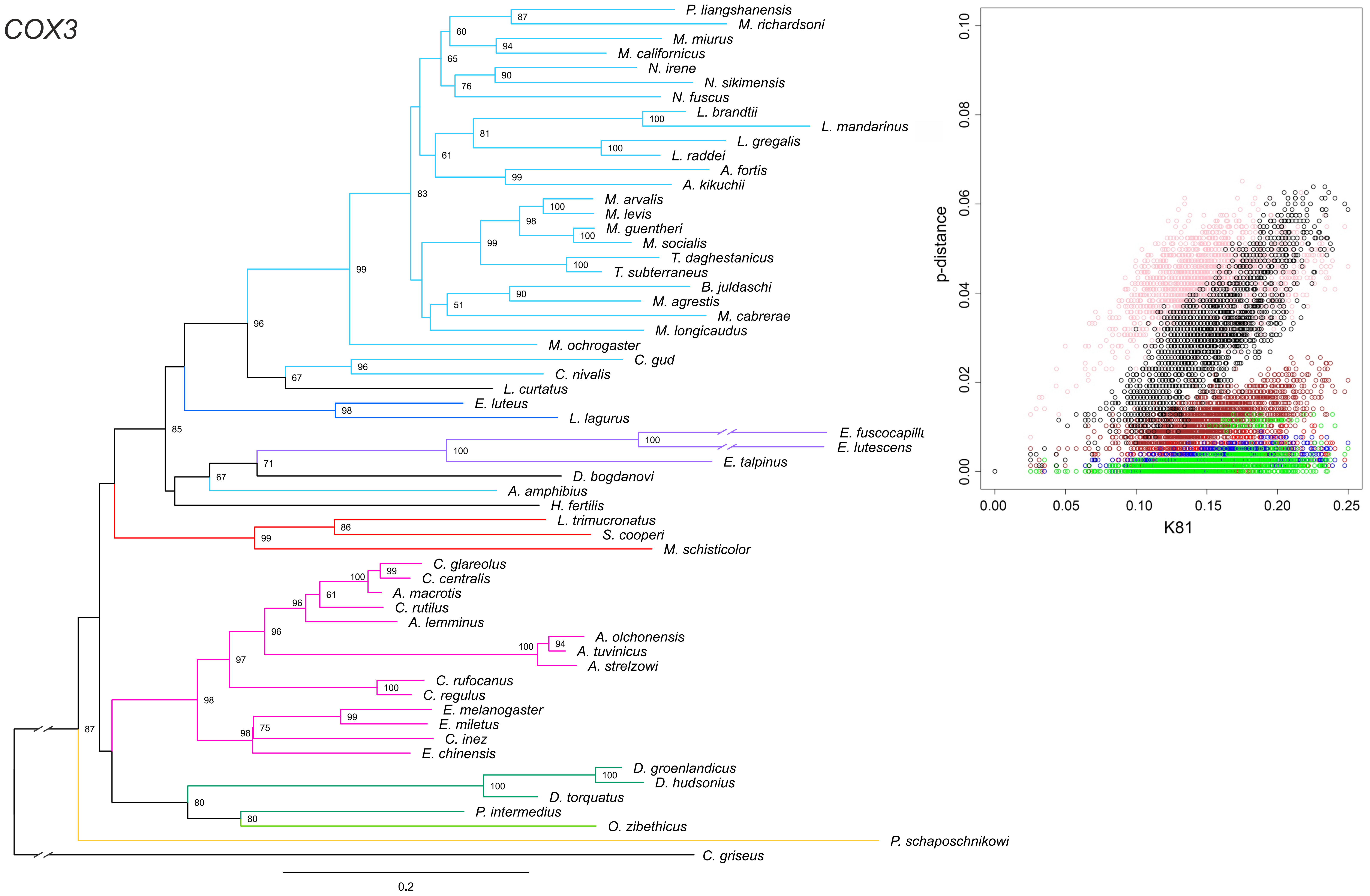

COX3
P. liangshanensis
M. richardsoni
M. miurus
M. californicus
N. irene
N. sikimensis
N. fuscus
L. brandtii
L. mandarinus
L. gregalis
L. raddei
A. fortis
A. kikuchii
M. arvalis
M. levis
M. guentheri
M. socialis
T. daghestanicus
T. subterraneus
B. juldaschi
M. agrestis
M. cabrerae
M. longicaudus
M. ochrogaster
C. gud
C. nivalis
L. curtatus
E. luteus
L. lagurus
E. fuscocapillu
E. lutescens
E. talpinus
D. bogdanovi
A. amphibius
H. fertilis
L. trimucronatus
S. cooperi
M. schisticolor
C. glareolus
C. centralis
A. macrotis
C. rutilus
A. lemminus
A. olchonensis
A. tuvinicus
A. strelzowi
C. rufocanus
C. regulus
E. melanogaster
E. miletus
C. inez
E. chinensis
D. groenlandicus
D. hudsonius
D. torquatus
P. intermedius
O. zibethicus
P. schaposchnikowi
C. griseus
0.2
p-distance
K81
0.00
0.05
0.10
0.15
0.20
0.25
0.02
0.04
0.06
0.08

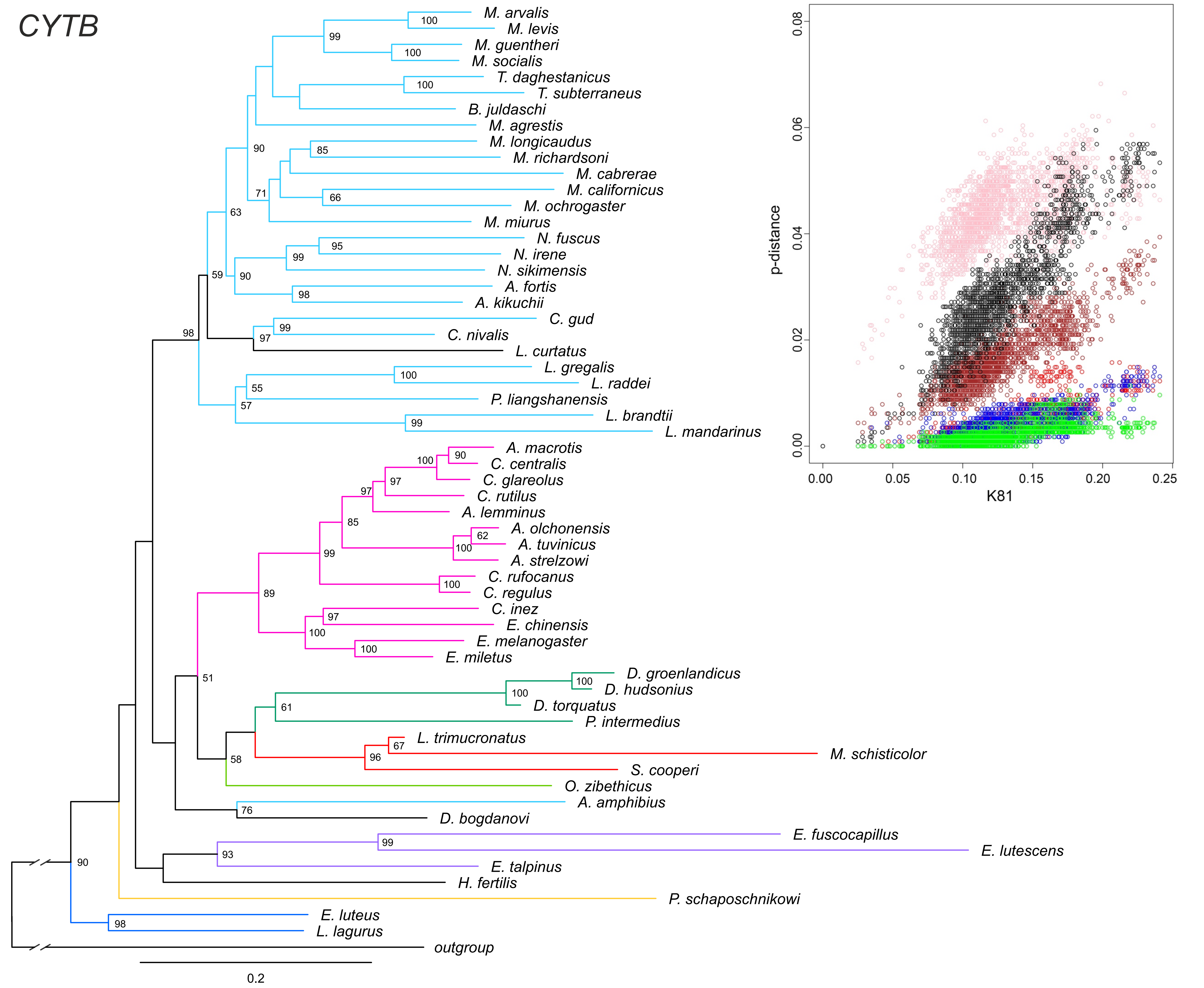
CYTB
M. arvalis
M. levis
M. guentheri
M. socialis
T. daghestanicus
T. subterraneus
B. juldaschi
M. agrestis
M. longicaudus
M. richardsoni
M. cabrerae
M. californicus
M. ochrogaster
M. miurus
N. fuscus
N. irene
N. sikimensis
A. fortis
A. kikuchii
C. gud
C. nivalis
L. curtatus
L. gregalis
L. raddei
P. liangshanensis
L. brandtii
L. mandarinus
A. macrotis
C. centralis
C. glareolus
C. rutilus
A. lemminus
A. olchonensis
A. tuvinicus
A. strelzowi
C. rufocanus
C. regulus
C. inez
E. chinensis
E. melanogaster
E. miletus
D. groenlandicus
D. hudsonius
D. torquatus
P. intermedius
L. trimucronatus
M. schisticolor
S. cooperi
O. zibethicus
A. amphibius
D. bogdanovi
E. fuscocapillus
E. lutescens
E. talpinus
H. fertilis
P. schaposchnikowi
E. luteus
L. lagurus
outgroup
0.2
p-distance
K81
0.00
0.02
0.04
0.06
0.08
0.00
0.05
0.10
0.15
0.20
0.25

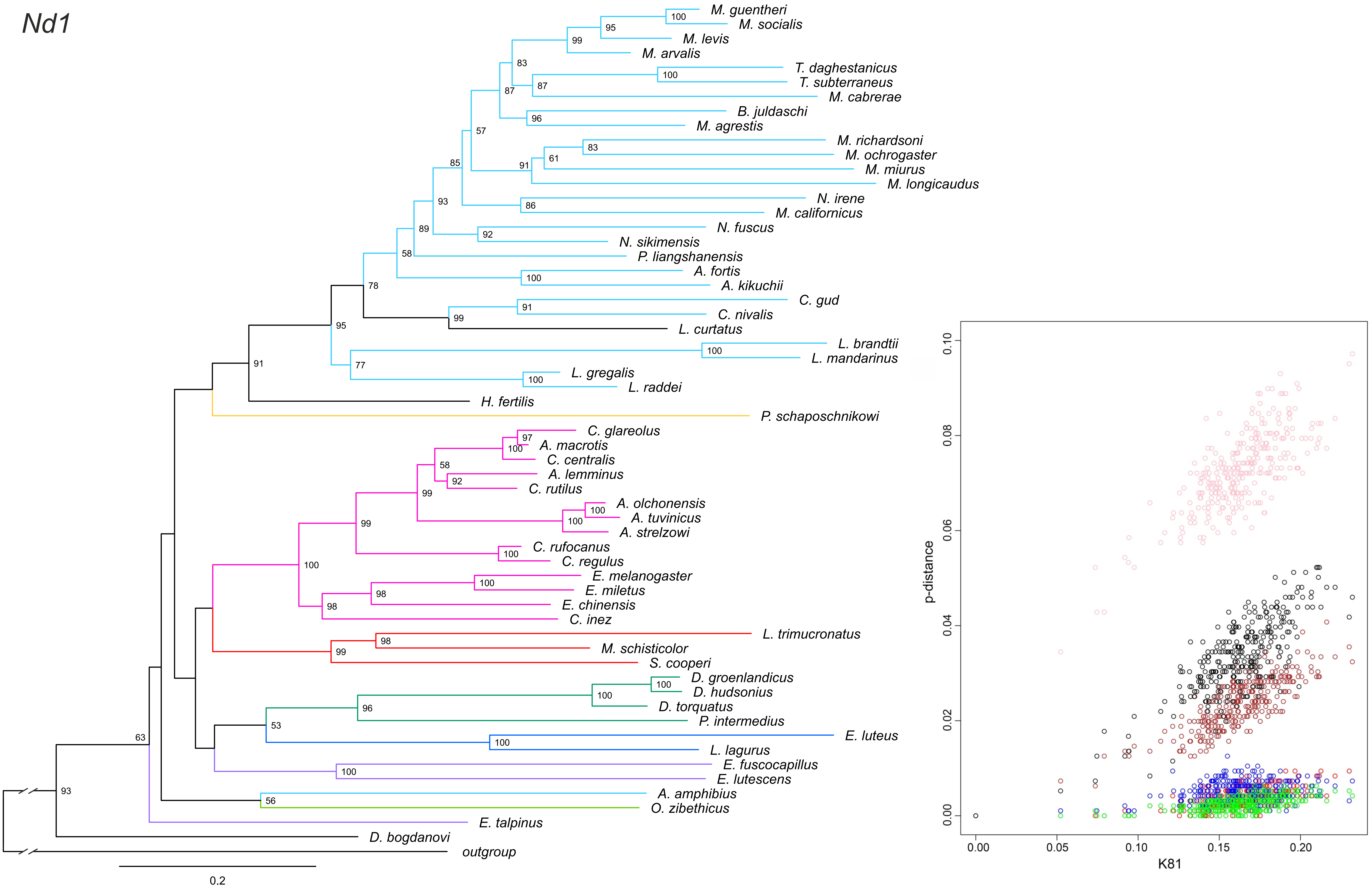

Nd1
M. guentheri
M. socialis
M. levis
M. arvalis
T. daghestanicus
T. subterraneus
M. cabrerae
B. juldaschi
M. agrestis
M. richardsoni
M. ochrogaster
M. miurus
M. longicaudus
N. irene
M. californicus
N. fuscus
N. sikimensis
P. liangshanensis
A. fortis
A. kikuchii
C. gud
C. nivalis
L. curtatus
L. brandtii
L. mandarinus
L. gregalis
L. raddei
H. fertilis
P. schaposchnikowi
C. glareolus
A. macrotis
C. centralis
A. lemminus
C. rutilus
A. olchonensis
A. tuvinicus
A. strelzowi
C. rufocanus
C. regulus
E. melanogaster
E. miletus
E. chinensis
C. inez
L. trimucronatus
M. schisticolor
S. cooperi
D. groenlandicus
D. hudsonius
D. torquatus
P. intermedius
E. luteus
L. lagurus
E. fuscocapillus
E. lutescens
A. amphibius
O. zibethicus
E. talpinus
D. bogdanovi
outgroup
0.2
p-distance
K81
0.00
0.02
0.04
0.06
0.08
0.10
0.00
0.05
0.10
0.15
0.20

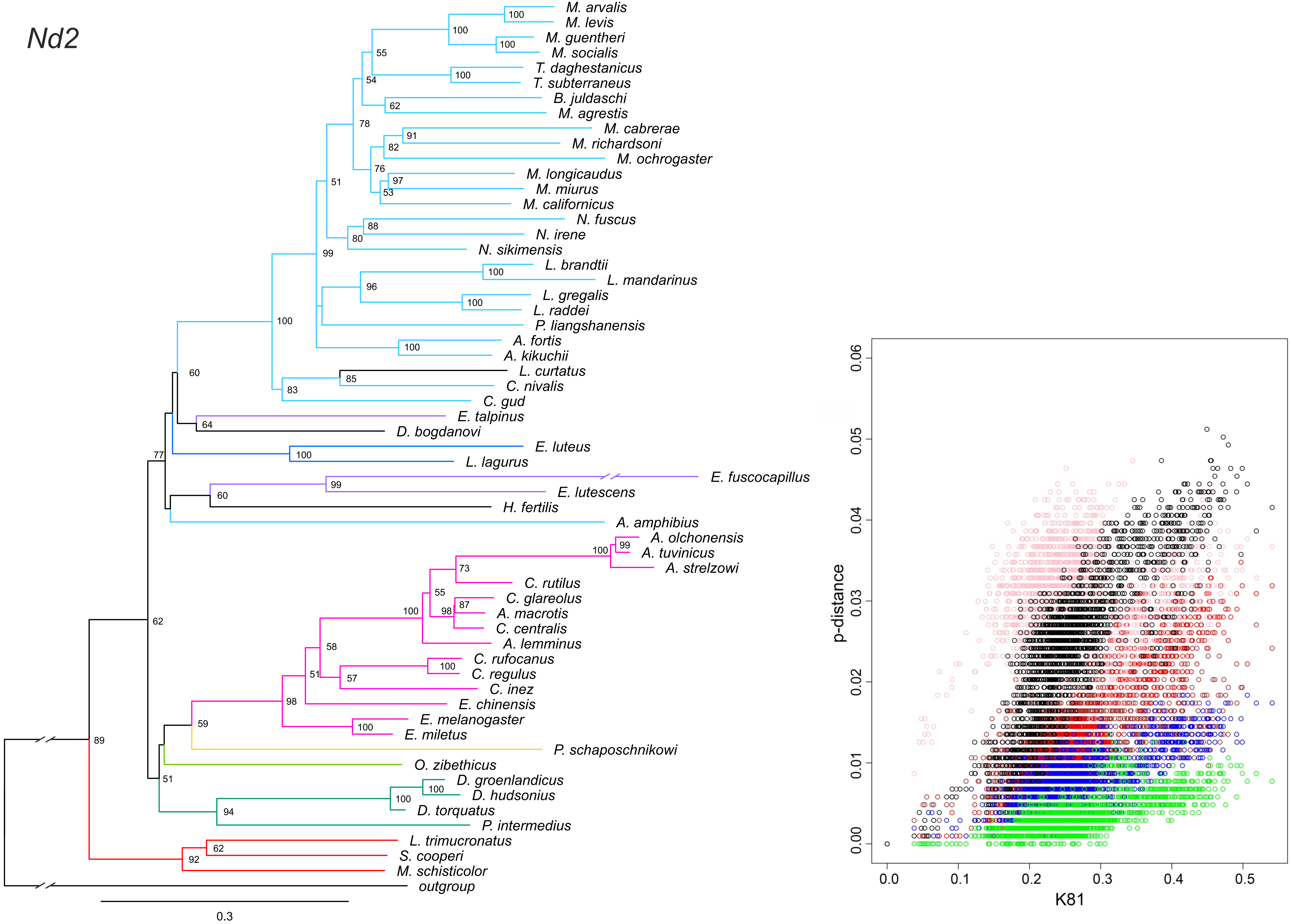

Nd2
M. arvalis
M. levis
M. guentheri
M. socialis
T. daghestanicus
T. subterraneus
B. juldaschi
M. agrestis
M. cabrerae
M. richardsoni
M. ochrogaster
M. longicaudus
M. miurus
M. californicus
N. fuscus
N. irene
N. sikimensis
L. brandtii
L. mandarinus
L. gregalis
L. raddei
P. liangshanensis
A. fortis
A. kikuchii
L. curtatus
C. nivalis
C. gud
E. talpinus
D. bogdanovi
E. luteus
L. lagurus
E. fuscocapillus
E. lutescens
H. fertilis
A. amphibius
A. olchonensis
A. tuvinicus
A. strelzowi
C. rutilus
C. glareolus
A. macrotis
C. centralis
A. lemminus
C. rufocanus
C. regulus
C. inez
E. chinensis
E. melanogaster
E. miletus
P. schaposchnikowi
O. zibethicus
D. groenlandicus
D. hudsonius
D. torquatus
P. intermedius
L. trimucronatus
S. cooperi
M. schisticolor
outgroup
0.3
p-distance
K81

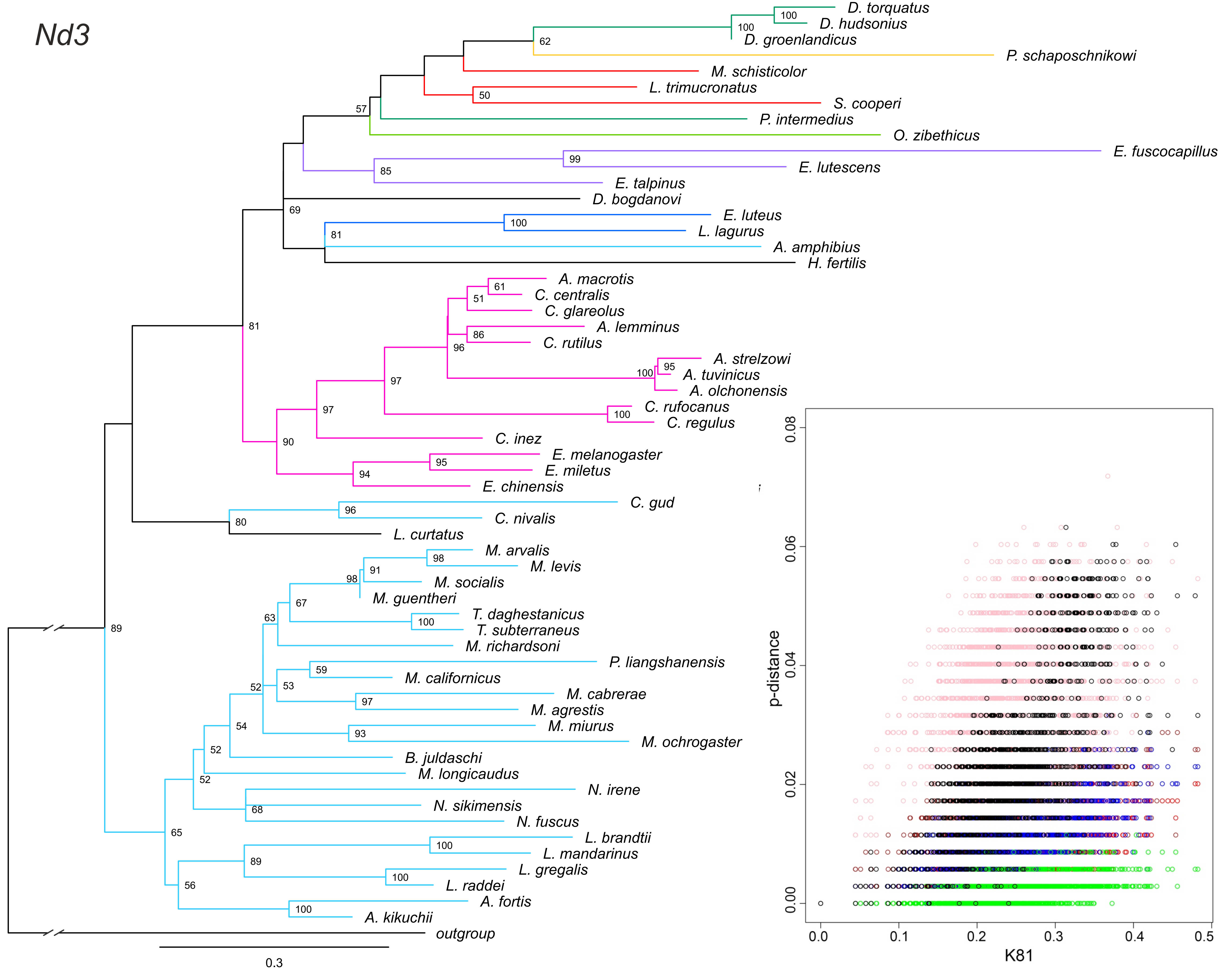

Nd3
D. torquatus
D. hudsonius
D. groenlandicus
P. schaposchnikowi
M. schisticolor
L. trimucronatus
S. cooperi
P. intermedius
O. zibethicus
E. fuscocapillus
E. lutescens
E. talpinus
D. bogdanovi
E. luteus
L. lagurus
A. amphibius
H. fertilis
A. macrotis
C. centralis
C. glareolus
A. lemminus
C. rutilus
A. strelzowi
A. tuvinicus
A. olchonensis
C. rufocanus
C. regulus
C. inez
E. melanogaster
E. miletus
E. chinensis
C. gud
C. nivalis
L. curtatus
M. arvalis
M. levis
M. socialis
M. guentheri
T. daghestanicus
T. subterraneus
M. richardsoni
P. liangshanensis
M. californicus
M. cabrerae
M. agrestis
M. miurus
M. ochrogaster
B. juldaschi
M. longicaudus
N. irene
N. sikimensis
N. fuscus
L. brandtii
L. mandarinus
L. gregalis
L. raddei
A. fortis
A. kikuchii
outgroup
0.3
p-distance
K81
0.00
0.02
0.04
0.06
0.08
0.0
0.1
0.2
0.3
0.4
0.5

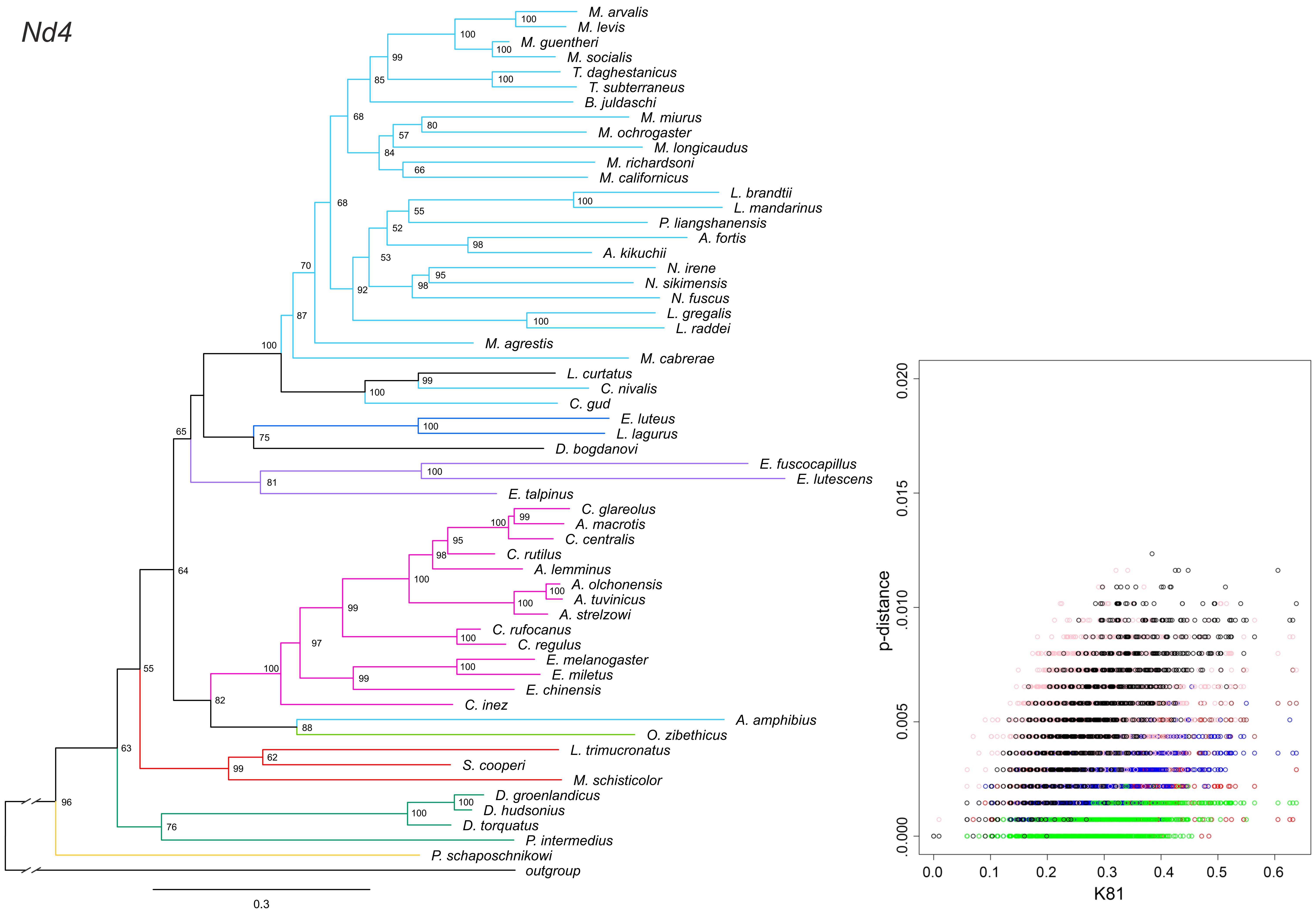
Nd4
M. arvalis
M. levis
M. guentheri
M. socialis
T. daghestanicus
T. subterraneus
B. juldaschi
M. miurus
M. ochrogaster
M. longicaudus
M. richardsoni
M. californicus
L. brandtii
L. mandarinus
P. liangshanensis
A. fortis
A. kikuchii
N. irene
N. sikimensis
N. fuscus
L. gregalis
L. raddei
M. agrestis
M. cabrerae
L. curtatus
C. nivalis
C. gud
E. luteus
L. lagurus
D. bogdanovi
E. fuscocapillus
E. lutescens
E. talpinus
C. glareolus
A. macrotis
C. centralis
C. rutilus
A. lemminus
A. olchonensis
A. tuvinicus
A. strelzowi
C. rufocanus
C. regulus
E. melanogaster
E. miletus
E. chinensis
C. inez
A. amphibius
O. zibethicus
L. trimucronatus
S. cooperi
M. schisticolor
D. groenlandicus
D. hudsonius
D. torquatus
P. intermedius
P. schaposchnikowi
outgroup
0.3
p-distance
0.000
0.005
0.010
0.015
0.020
K81
0.0
0.1
0.2
0.3
0.4
0.5
0.6

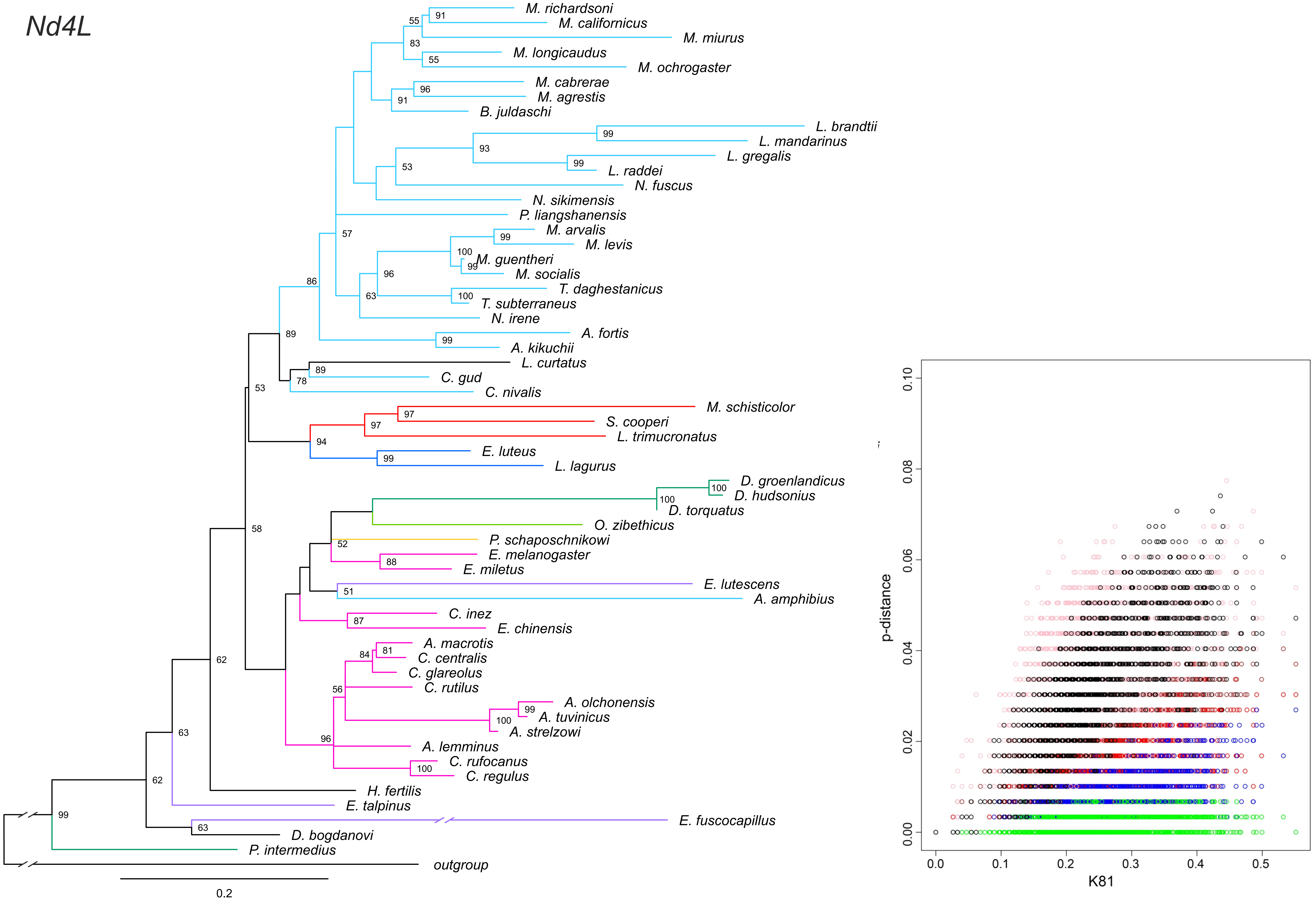
Nd4L
M. richardsoni
M. californicus
M. miurus
M. longicaudus
M. ochrogaster
M. cabrerae
M. agrestis
B. juldaschi
L. brandtii
L. mandarinus
L. gregalis
L. raddei
N. fuscus
N. sikimensis
P. liangshanensis
M. arvalis
M. levis
M. guentheri
M. socialis
T. daghestanicus
T. subterraneus
N. irene
A. fortis
A. kikuchii
L. curtatus
C. gud
C. nivalis
M. schisticolor
S. cooperi
L. trimucronatus
E. luteus
L. lagurus
D. groenlandicus
D. hudsonius
D. torquatus
O. zibethicus
P. schaposchnikowi
E. melanogaster
E. miletus
E. lutescens
A. amphibius
C. inez
E. chinensis
A. macrotis
C. centralis
C. glareolus
C. rutilus
A. olchonensis
A. tuvinicus
A. strelzowi
A. lemminus
C. rufocanus
C. regulus
H. fertilis
E. talpinus
E. fuscocapillus
D. bogdanovi
P. intermedius
outgroup
0.2
p-distance
K81
0.00
0.02
0.04
0.06
0.08
0.10
0.0
0.1
0.2
0.3
0.4
0.5

Nd5

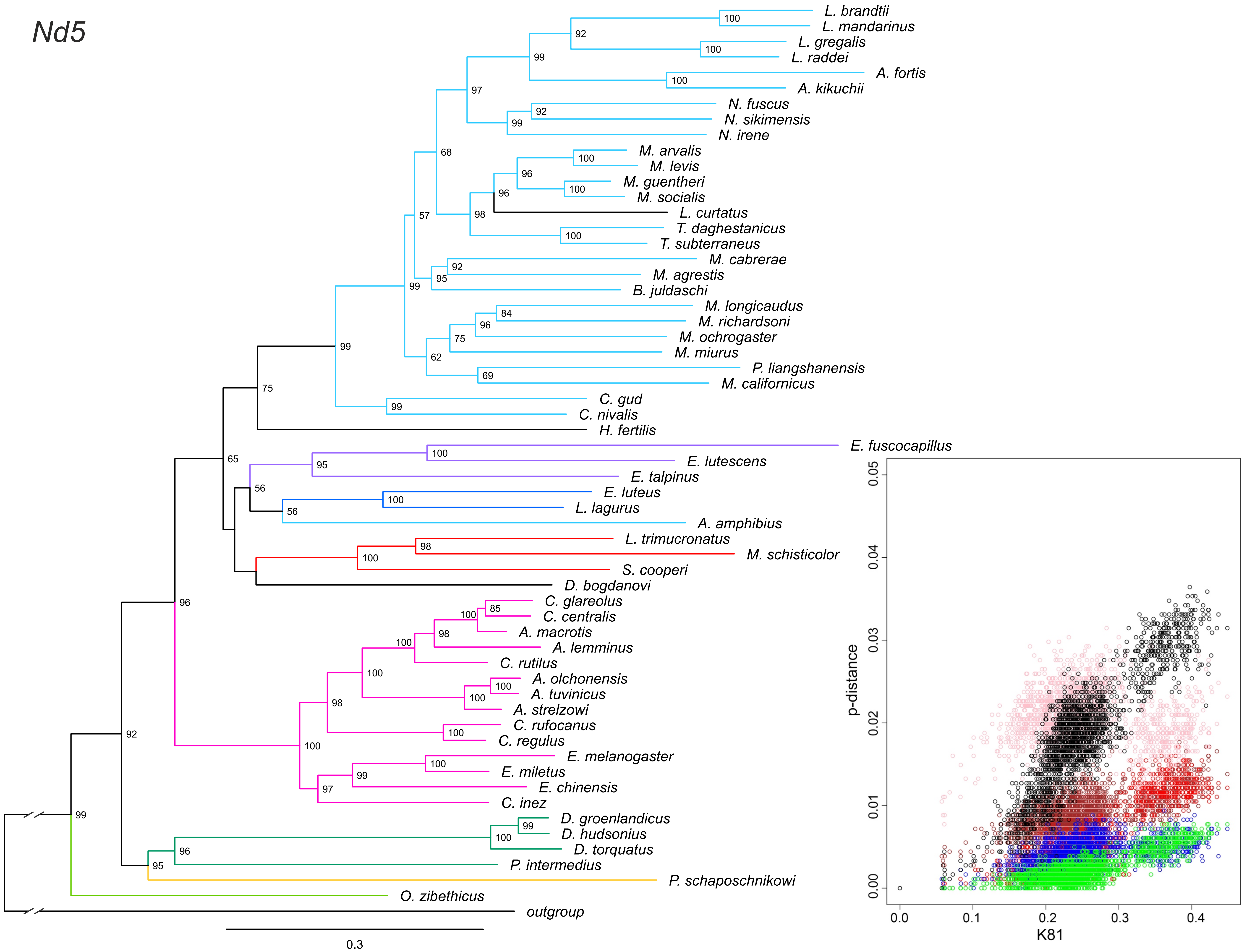

L. brandtii
L. mandarinus
L. gregalis
L. raddei
A. fortis
A. kikuchii
N. fuscus
N. sikimensis
N. irene
M. arvalis
M. levis
M. guentheri
M. socialis
L. curtatus
T. daghestanicus
T. subterraneus
M. cabrerae
M. agrestis
B. juldaschi
M. longicaudus
M. richardsoni
M. ochrogaster
M. miurus
P. liangshanensis
M. californicus
C. gud
C. nivalis
H. fertilis
E. fuscocapillus
E. lutescens
E. talpinus
E. luteus
L. lagurus
A. amphibius
L. trimucronatus
M. schisticolor
S. cooperi
D. bogdanovi
C. glareolus
C. centralis
A. macrotis
A. lemminus
C. rutilus
A. olchonensis
A. tuvinicus
A. strelzowi
C. rufocanus
C. regulus
E. melanogaster
E. miletus
E. chinensis
C. inez
D. groenlandicus
D. hudsonius
D. torquatus
P. intermedius
P. schaposchnikowi
O. zibethicus
outgroup
0.3
p-distance
K81
0.00
0.01
0.02
0.03
0.04
0.05
0.0
0.1
0.2
0.3
0.4

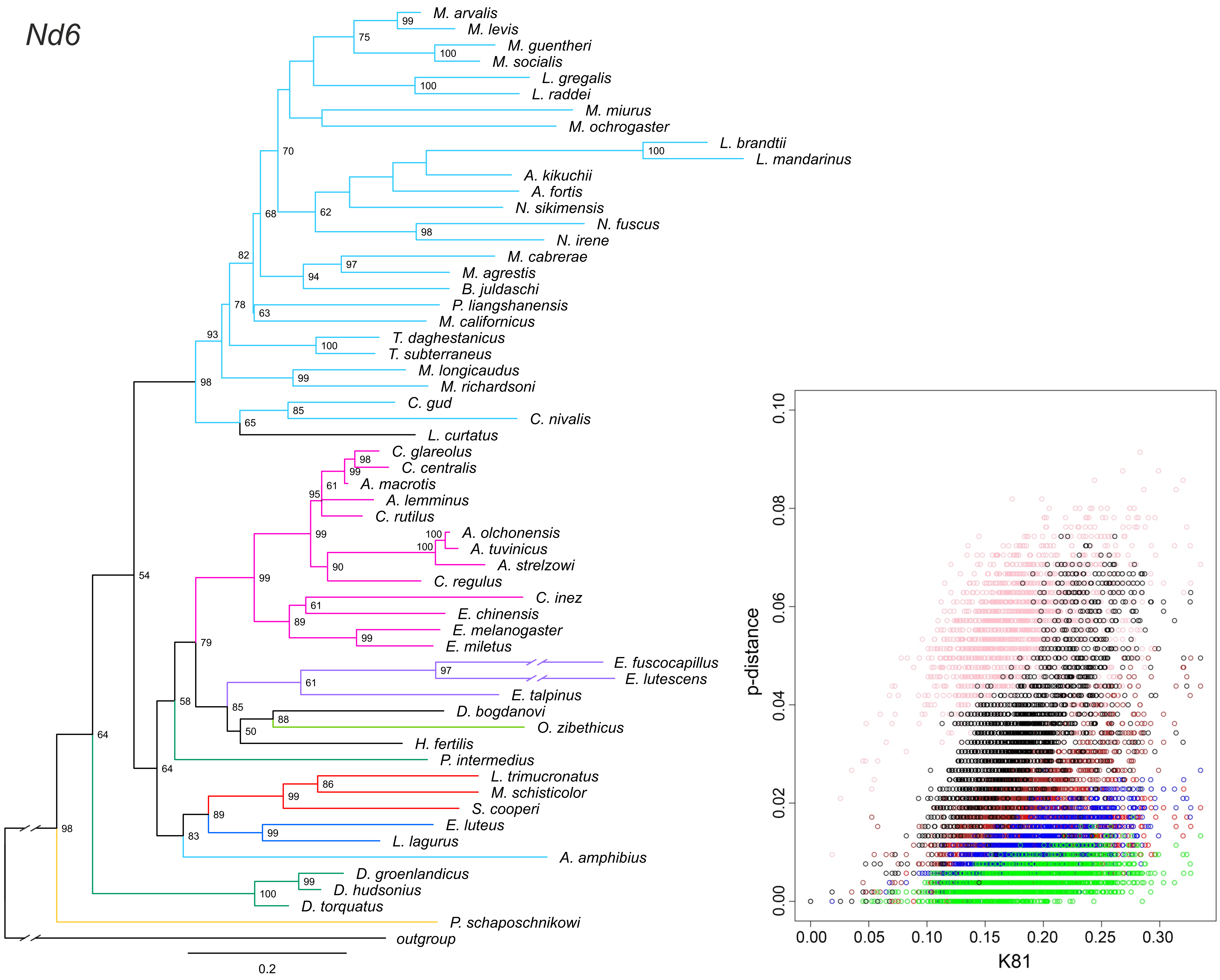
Nd6
M. arvalis
M. levis
M. guentheri
M. socialis
L. gregalis
L. raddei
M. miurus
M. ochrogaster
L. brandtii
L. mandarinus
A. kikuchii
A. fortis
N. sikimensis
N. fuscus
N. irene
M. cabrerae
M. agrestis
B. juldaschi
P. liangshanensis
M. californicus
T. daghestanicus
T. subterraneus
M. longicaudus
M. richardsoni
C. gud
C. nivalis
L. curtatus
C. glareolus
C. centralis
A. macrotis
A. lemminus
C. rutilus
A. olchonensis
A. tuvinicus
A. strelzowi
C. regulus
C. inez
E. chinensis
E. melanogaster
E. miletus
E. fuscocapillus
E. lutescens
E. talpinus
D. bogdanovi
O. zibethicus
H. fertilis
P. intermedius
L. trimucronatus
M. schisticolor
S. cooperi
E. luteus
L. lagurus
A. amphibius
D. groenlandicus
D. hudsonius
D. torquatus
P. schaposchnikowi
outgroup
0.2
p-distance
K81
0.00
0.02
0.04
0.06
0.08
0.10
0.00
0.05
0.10
0.15
0.20
0.25
0.30
