# Supplementary material for: A mitochondrial genome phylogeny of voles and lemmings (Rodentia: Arvicolinae): Evolutionary and taxonomic implications
Source: PLoS One. 2021 Nov 19;16(11):e0248198. doi: 10.1371/journal.pone.0248198 (PMC8604340; doi:10.1371/journal.pone.0248198)
Supplement: S2 File — (DOCX) [file pone.0248198.s012.docx]

**S2 File. The proposed system of generic group taxa within the tribe Arvicolini *sensu stricto***

Genus *Hyperacrius*

Genus *Lemmiscus*

Genus *Chionomys*

Genus *Proedromys*

Genus *Neodon*

Genus *Alexandromys*

Genus *Lasiopodomys*

Subgenus *Lasiopodomys*

Subgenus *Stenocranius*

Genus *Mynomes*

Genus *Blanfordimys*

Subgenus *Blanfordimys*

Subgenus *Agricola*

Subgenus *Iberomys*

Genus *Terricola*

Genus *Microtus*

Subgenus *Microtus*

Subgenus *Sumeriomys*
